# Midterm Outcomes of Medial Patellofemoral Ligament Reconstruction in Adolescent Athletes: Comparison Between Acute and Recurrent Patella Dislocation

**DOI:** 10.3390/jcm14144881

**Published:** 2025-07-09

**Authors:** Georgios Kalinterakis, Christos K. Yiannakopoulos, Christos Koukos, Konstantinos Mastrantonakis, Efstathios Chronopoulos

**Affiliations:** 1 School of Physical Education and Sport Science, National and Kapodistrian University of Athens, 17237 Athens, Greece; 2 Laboratory for Research of the Musculoskeletal System “Th. Garofalidis”, Medical School, National and Kapodistrian University of Athens, KAT General Hospital, Kifissia, 14561 Athens, Greece

**Keywords:** knee, medial patellofemoral ligament, patella dislocation, adolescence

## Abstract

**Background/Objectives**: Patellar instability in adolescents is a significant cause of short- and long-term morbidity and disability. Traditionally, patients with first-time patellar dislocation are managed nonoperatively, although most studies are not focusing on the adolescent athletic population. The primary objective of the current study was to compare patient-reported outcomes and complications in adolescent athletes who underwent surgery either after the first patellar dislocation or after the recurrence of the dislocation with a minimum postoperative follow-up of 48 months (48–75 months). **Methods:** A total of 39 adolescent athletes who underwent medial patellofemoral ligament (MPFL) reconstruction (Group A, after the first dislocation, and Group B, recurrent patella dislocation) were included in this study. In all the patients, the same MPFL reconstruction technique was applied using a semitendinosus autograft. The graft was fixed on the patella using a transverse tunnel and adjustable loop button fixation and, in the femur, using a tunnel and absorbable screw fixation. The tunnel was drilled obliquely to prevent penetration of the distal femoral physis. The preoperative and postoperative clinical and functional evaluations of the patients were conducted via the visual analog scale (VAS), the Lysholm Knee Scoring System, the Kujala Anterior Knee Pain Scale, and the Pediatric International Knee Documentation Committee (Pedi-IKDC), and the return to sports score was assessed via the Tegner Activity Scale (TAS). **Results:** At the latest follow-up, both groups demonstrated significant improvement in the Lysholm scores, with Group A achieving a mean of 92.57 ± 6.21 and Group B achieving a mean of 90.53 ± 8.21 (*p* = 0.062). Postoperatively, Group A achieved a mean Kujala score of 94.21 ± 9.23, whereas Group B reached 92.76 ± 12.39, with no statistically significant difference (*p* = 0.08). The Pedi-IKDC score improved postoperatively in both groups. In Group A, it increased from 67.98 ± 12.29 to 93.65 ± 4.1, and in Group B, from 56.21 ± 13.6 to 91.67 ± 6.21 (*p* = 0.067). The preoperative visual analog scale (VAS) score for pain was significantly lower in Group A (3.1 ± 1.13) than in Group B (4.2 ± 3.01, *p* < 0.01). At the latest follow-up, the VAS scores improved in both groups, with Group A reporting a mean score of 0.47 ± 1.01 and Group B 0.97 ± 1.32 (*p* = 0.083). The Tegner activity scores were similar between the groups preoperatively, with Group A at 7.72 ± 1.65 and Group B at 7.45 ± 2.09 (*p* = 0.076). Postoperatively, Group A had a mean score of 7.28 ± 2.15, whereas Group B had a mean score of 6.79 ± 3.70 (*p* = 0.065). The mean time to return to sports was significantly shorter in Group A (5.1 ± 1.3 months) than in Group B (7.6 ± 2.1 months) (*p* < 0.01). Overall, 84.61% of the patients returned to their previous activity level. Specifically, 95.2% (20/21) of patients in Group A achieved this outcome, whereas 72.22% (13/18) achieved it in Group B. Patient satisfaction was generally high, with 76% (16/21) of patients in Group A reporting being satisfied or very satisfied, compared with 77% (14/18) in Group B. **Conclusions:** MPFL reconstruction is a safe and effective procedure for both acute and recurrent patellar dislocation in adolescent athletes. While patients who underwent acute reconstruction returned to sport more quickly and showed higher absolute postoperative scores, the greatest overall improvement from preoperative to final follow-up was observed in those treated for recurrent instability. Both surgical approaches demonstrated high satisfaction rates and minimal complications, supporting MPFL reconstruction as a reliable option in both acute and recurrent cases.

## 1. Introduction

Patellar instability in adolescents is a significant orthopedic injury that can lead to considerable short- and long-term disability. The incidence of patellar dislocation in this age group is estimated to be nearly six times higher than that in the general population, with reported rates of 29 per 100,000 compared with 5.8 per 100,000, respectively [1,2]. In children and adolescents who experience first-time patellar dislocation and undergo conservative treatment, the recurrence rate may be as high as 34% [3]. Traditionally, patients with first-time patellar dislocation have been managed nonoperatively, as low recurrence rates and high success rates are generally anticipated with conservative treatment. However, benign neglect may not be an appropriate approach, especially in certain patient populations, including athletes and patients engaging in regular athletic activities. Newer evidence suggests that younger age, the presence of an open physis, and torsional abnormality contribute to recurrence, causing disability, pain, and poor function [4].

The medial patellofemoral ligament (MPFL) is recognized as a key structure in stabilizing the patella, preventing lateral dislocation, and serving as the primary restraint against lateral patellar displacement [5]. Reconstruction of the MPFL has been proven to prevent lateral patellar dislocation, with several reports of favorable outcomes in the treatment of pediatric patients [6,7,8]. In patients with open distal femoral physes, MPFL reconstruction has been shown to be safe, with an acceptable risk of complications [9,10]. Consequently, there has been a paradigm shift toward surgical treatment of patients with first-time patellar dislocation, including the pediatric population [11].

To the best of our knowledge, no study has compared the outcomes of MPFL reconstruction between acute and recurrent patellar dislocations in adolescent athletes. Therefore, the primary objective of the current study was to compare the patient-reported outcomes and complications in adolescent athletes who underwent surgery either after the first patellar dislocation or after the recurrence of the dislocation. The null hypothesis was that there would be no difference in postoperative complication rates, return-to-sport rates, or patient-reported outcomes between patients who underwent MPFL reconstruction for acute versus recurrent patella instability.

## 2. Materials and Methods

This was a retrospective review of prospectively collected data from a cohort of 39 adolescent athletes operated on between January 2019 and May 2022. All surgeries were carried out at the same hospital by a senior surgeon (C.K.Y.), and the minimum postoperative follow-up was 48 months (48–75 months). All patients and their parents were fully informed about the study, and written consent for their participation was obtained prior to the operation. The study was approved by the Institutional Research Board (Approval Number 80/1-09-2021).

The inclusion criteria for the study were as follows: (1) adolescent athletes (12–17 years old) and (2) one or more documented episodes of patellar dislocation. The exclusion criteria were as follows: (1) adult patients with a closed physis; (2) patients with a history of ipsilateral knee surgery; (3) patients with underlying neurological and hypermobility syndromes that affected neuromuscular performance; (4) patients with intellectual or physical disabilities; (5) patients with severe accompanying injuries, such as ACL injury or osteochondral fractures necessitating open or extensive surgery; and (6) athletes with severe lower limb rotational disorders (just 1 case).

The participants in this cohort were divided into two groups on the basis of the timing of surgery following their first patellar dislocation. Group A included patients who underwent surgery after the first dislocation episode, whereas Group B included patients who underwent surgery for recurrent patellar instability with more than 3 dislocation episodes. Patients were classified as athletes if they were actively participating in organized sports at the time of injury, defined as a Tegner activity level ≥ 6. This included regular participation in school, club, or regional sports competitions with structured training. Additionally, patients were radiologically evaluated with standing AP and lateral radiographs and axial patellar views. MRI was performed on all patients to assess trochlear dysplasia and intra-articular pathology. Any detected trochlear dysplasia was documented, its significance was explained to the patients and their parents, and it was benignly neglected, as only soft-tissue procedures are indicated in the adolescent population because of the presence of an open physis.

Preoperative and postoperative clinical and functional evaluations of the patients were conducted via the visual analog scale (VAS), the Lysholm Knee Scoring System, the Kujala Anterior Knee Pain Scale, and the Pediatric International Knee Documentation Committee (Pedi-IKDC), and the return to sports score was assessed via the Tegner Activity Scale (TAS). The VAS is a well-established tool for self-reporting acute pain in children. It has been validated and is recommended for use in children aged 8–17 years [12]. The Kujala Anterior Knee Pain Scale is widely used for the assessment of patellofemoral disorders, taking into account different aspects such as pain, stiffness, swelling, and limitations in activities such as climbing or squatting [13]. The Lysholm scale assesses symptoms and complaints, as well as functioning in daily activities to a limited extent, but it does not evaluate the domain of functioning in sports and recreational activities. It has been shown to be a valid and reliable questionnaire in adolescents (12–17 years) [14]. The Pedi-IKDC is an adapted version of the International Knee Documentation Committee’s scale, which is specifically tailored for younger patients [15]. Finally, the Tegner activity scale has been used in the pediatric population, although younger patients may sometimes have difficulty understanding it [16]. In skeletally immature patients, the possibility of skeletal growth disturbance was assessed through a combination of clinical and imaging evaluations by two different physicians.

**Figure 1:**
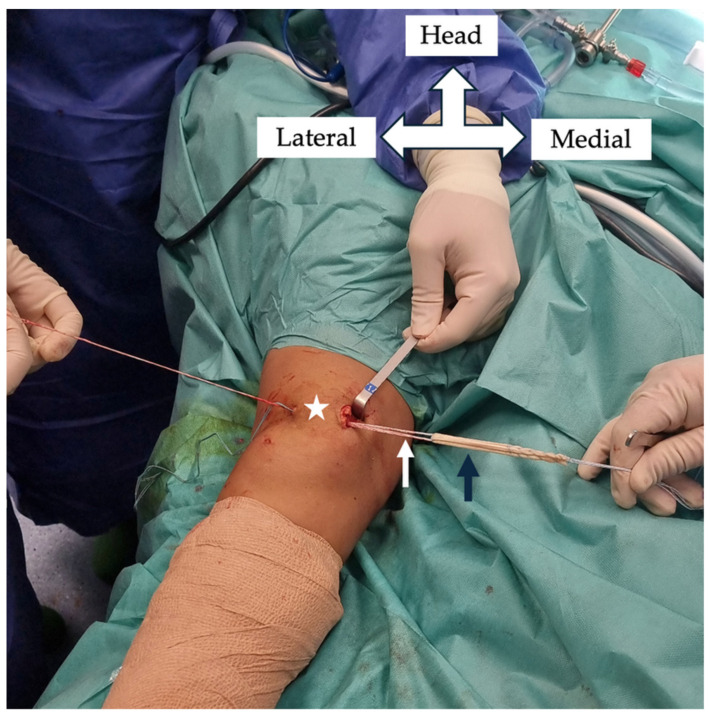
Intraoperative photograph showing the passage of the semitendinosus autograft (black arrow) through the patellar tunnel (star) using an adjustable cortical fixation button (white arrow).

All operations were performed under general anesthesia and tourniquet ischemia in the supine position. Standard diagnostic arthroscopy was initially performed in all patients to evaluate patella tracking and diagnose and treat any intra-articular pathology. The graft selected for the procedure was the semitendinosus tendon, which was harvested via a posterior mini approach to minimize aesthetic concerns when a tendon stripper was used. The superomedial border of the patella was approached with a mini vertical incision 2–3 cm in length. The graft was secured in the patella with an adjustable loop device. Specifically, a guide pin was inserted into the patella under fluoroscopic guidance, starting at its proximal medial edge and penetrating the lateral patellar cortex. A 4 mm cannulated drill was used to breach the lateral patellar cortex to facilitate passage and flipping of the adjustable loop button. A 20 mm long, partial-width patella tunnel was then drilled via a 6 mm cannulated drill to facilitate passage of the looped graft (Figure 1).

**Figure 2:**
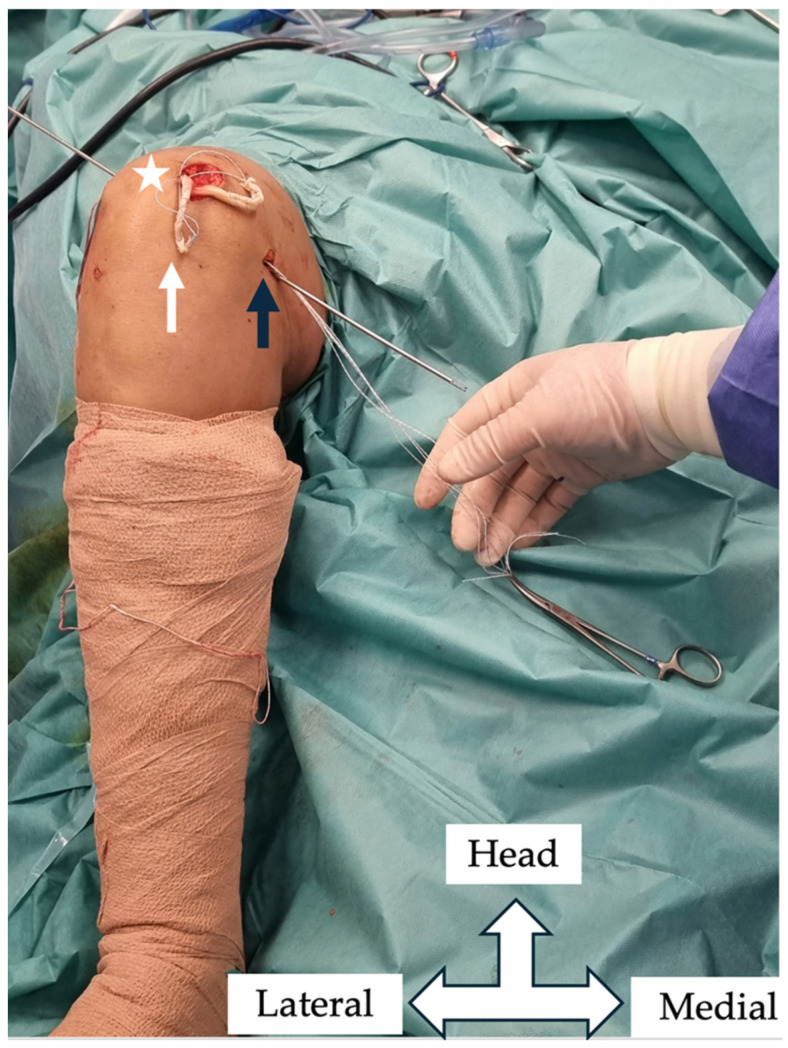
Intraoperative photograph. The semitendinosus autograft (white arrow) is inserted in a patellar (star) partial-width tunnel and passed through subcutaneously to the femoral fixation point (black arrow).

**Figure 3:**
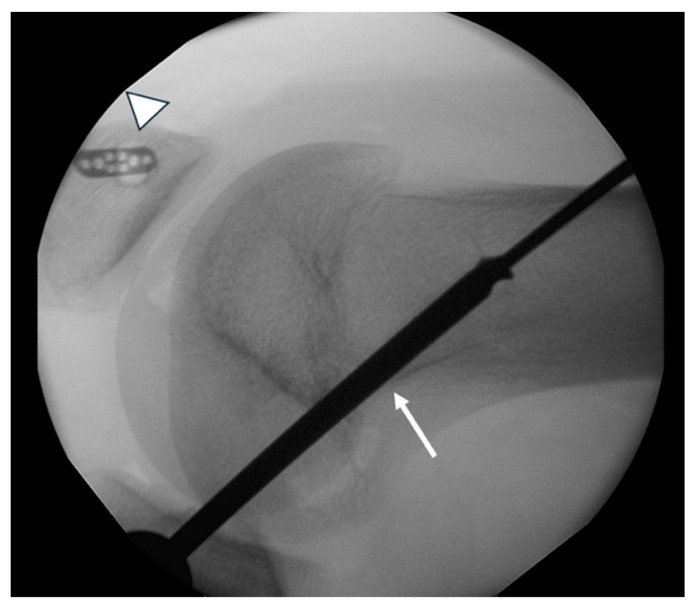
Lateral fluoroscopic view of the distal femur. The patella button fixation is shown (arrowhead). The femoral tunnel is created in a proximal–lateral direction (arrow) to avoid injuring the distal femoral physis.

**Figure 4:**
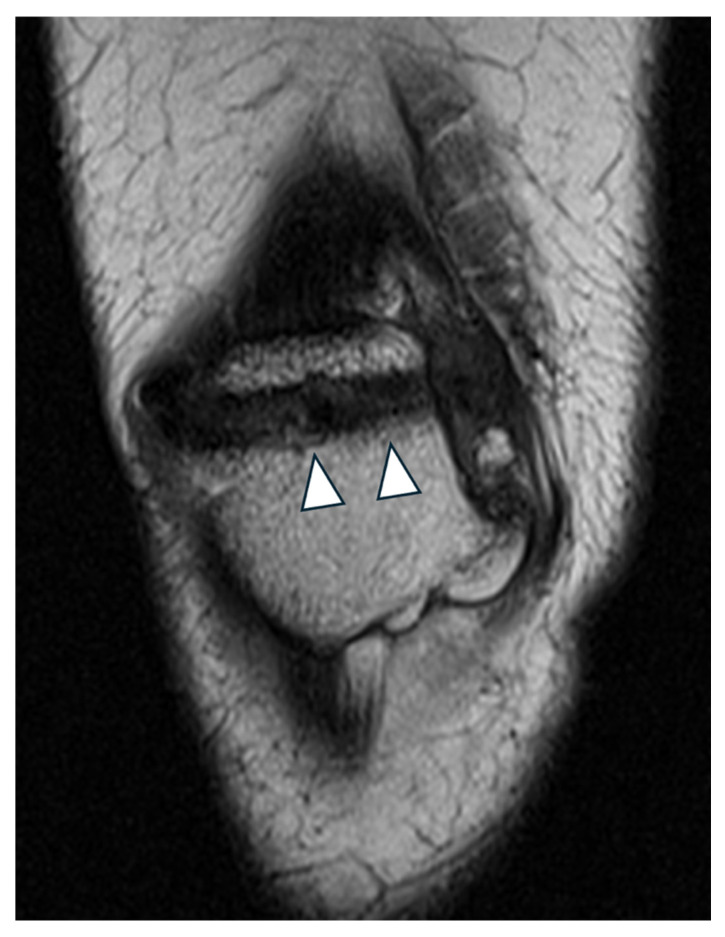
Coronal MRI of the patella in a patient with previous MPFL reconstruction. A semitendinosus graft was passed transversely through the patella (arrowheads) and fixed with an adjustable loop button (not shown).

**Figure 5:**
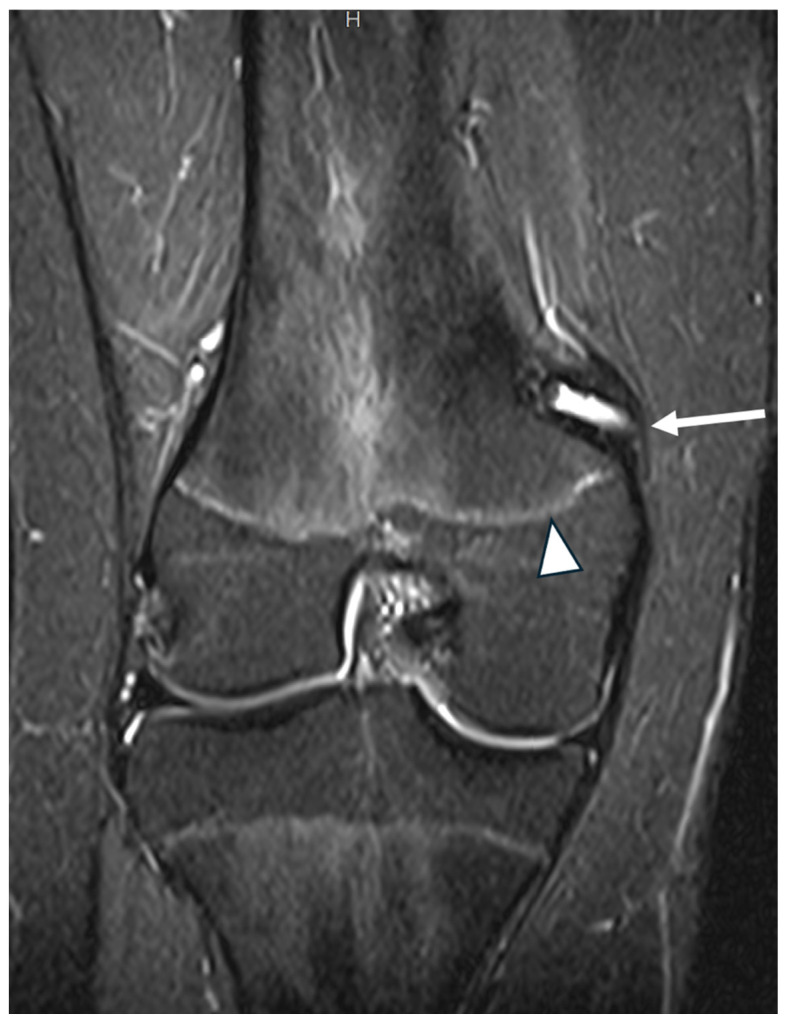
Coronal MRI of the distal femur in an adolescent. The femoral tunnel for the passage of the semitendinosus graft was created superior to the distal femoral physis (arrowhead) in a proximal direction to avoid physis penetration and fixed with an absorbable screw (arrow).

In the femur, the Schöttle point was located via fluoroscopic guidance to prevent interference with the growth plate. A guide pin was inserted obliquely in a superolateral direction to avoid penetration of the physis, and a 6 mm tunnel was created under fluoroscopic guidance (Figure 2 and Figure 3). The two ends of the semitendinosus graft were pulled into the femoral tunnel, and once isometry was determined, final graft fixation was performed with the knee flexed at 60 degrees using a 7 mm absorbable screw. Graft positioning is clearly shown in postoperative MRI imaging (Figure 4 and Figure 5).

No postoperative knee immobilization was used. The patients were instructed to bear weight using elbow crutches for 3 weeks and limit knee flexion to 60 degrees. Gradual increase in the knee range of motion during the following 3 weeks was allowed to avoid knee stiffness.

Following surgery, the importance of adhering to specific rehabilitation guidelines was emphasized. The rehabilitation protocol comprises 4 phases. Phase 1 (weeks 1–4) is focused on protection, reducing swelling, maintaining full knee extension, and regaining quadriceps control. Patients were encouraged to gradually increase knee flexion to 100° by the fourth week. Phase 2 (weeks 5–6) emphasizes improving strength and balance, with a goal of >100° knee flexion. Phase 3 (weeks 7–12) involves functional strengthening, with controlled exercises and gait correction. By Phase 4 (weeks 12–16), patients start light running, progressing to sports-specific drills by the 20th week. Return to sport was defined as the time from surgery to full participation in pre-injury sport activities, based on patient and/or parent report and confirmed during routine clinical follow-up. While the rehabilitation protocol was initiated, sport-specific drills were conducted at approximately 20 weeks, actual RTS was individualized based on clinical assessment and surgeon guidance.

Statistical analysis was performed using SPSS Statistics v29.0.2 (IBM Corp., Armonk, NY, USA). Continuous variables, such as the preoperative and postoperative values for the VAS, Kujala, Lysholm, TAS, and Pedi-IKDC scores, were reported as mean ± standard deviation (SD), and categorical variables as frequencies and percentages. Normality of continuous variables was tested using Shapiro–Wilk, and equality of variances using Levene’s test. Data following a normal distribution were compared using Student’s *t*-test, and those not normally distributed were compared using Mann–Whitney U. When analyzing data from paired samples, the *t*-test for paired samples or Wilcoxon rank-sum test was used. Categorical variables were compared using Chi-Square test or Fisher’s exact test. A *p*-value of <0.05 was considered statistically significant.

## 3. Results

### 3.1. Demographic Data

A total of 39 adolescent athletes underwent anatomical MPFL reconstruction. Among them, 21 patients underwent surgery after the first patellar dislocation (Group A), and 18 underwent surgery due to recurrent patellar dislocation (Group B). The demographic characteristics, including age, sex, and body mass index (BMI), were similar between the two groups, with no statistically significant differences observed (*p* > 0.05). Trochlear dysplasia was assessed according to the Dejour classification. In Group A, 16 patients were classified as type A, 3 as type B, 1 as type C, and 1 as type D. In Group B, 10 patients were classified as type A, 5 as type B, and 3 as type C. No type D dysplasia was observed in Group B. The most common sport associated with injury was football, accounting for 11 cases in Group A and 12 cases in Group B. Basketball-related injuries were the next most common, with eight cases in Group A and five cases in Group B. Other sports were represented by two athletes in Group A and one athlete in Group B. Patient characteristics are depicted in Table 1. The mean postoperative follow-up was 61 ± 9.74 months for Group A and 64 ± 11.68 months for Group B.

### 3.2. Clinical Outcomes

The mean preoperative Lysholm score for Group A was 74.18 ± 19.23, which was significantly greater than that for Group B, which had a mean score of 62.11 ± 11.65 (*p* < 0.01). At the latest follow-up, both groups demonstrated significant improvement in the Lysholm scores, with Group A achieving a mean of 92.57 ± 6.21 and Group B achieving a mean of 90.53 ± 8.21 (*p* = 0.062). The mean change (Δ) in the Lysholm score was 18.39 ± 20.25 for Group A and 28.42 ± 8.62 for Group B (*p* = 0.037). The Kujala score followed a similar trend. Preoperatively, Group A had a mean score of 68.21 ± 21.01, which was significantly greater than that of Group B (55.32 ± 17.09) (*p* < 0.01). Postoperatively, Group A achieved a mean Kujala score of 94.21 ± 9.23, whereas Group B reached 92.76 ± 12.39, with no statistically significant difference (*p* = 0.08). The corresponding mean change (Δ) in Kujala score was 26.00 ± 22.17 for Group A and 37.44 ± 21.51 for Group B (*p* = 0.046). The Pedi-IKDC score also differed preoperatively between the groups. Group A had a mean score of 67.98 ± 12.29, whereas Group B had a lower mean score of 56.21 ± 13.6 (*p* < 0.01). Postoperatively, Group A improved to 93.65 ± 4.1, and Group B reached 91.67 ± 6.21 (*p* = 0.067). The mean change (Δ) in Pedi-IKDC score was 25.67 ± 13.14 for Group A and 35.46 ± 13.13 for Group B (*p* = 0.006). The pre- and postoperative scores, as well as Δ values, are presented in Table 2 and Table 3.

### 3.3. VAS Score and Knee Range of Motion

The VAS score for pain was significantly lower in Group A (3.1 ± 1.13) than in Group B (4.2 ± 3.01, *p* < 0.01). At the latest follow-up, the VAS scores improved in both groups, with Group A reporting a mean score of 0.47 ± 1.01 and Group B 0.97 ± 1.32 (*p* = 0.083), although these differences were not statistically significant. The mean change (Δ) in VAS score was −2.63 ± 1.66 in Group A and −3.23 ± 2.49 in Group B (*p* = 0.238), indicating comparable improvements in perceived pain. With respect to the knee range of motion, Group A and Group B had comparable mean preoperative ranges of motion: 131° ± 8.29 and 130° ± 11.19, respectively (*p* = 0.09). At the latest follow-up, both groups showed a near-normal range of motion, with Group A achieving a mean of 132° ± 7.54 and Group B 133° ± 9.26 (*p* = 0.071). The pre- and postoperative VAS scores, as well as Δ values, are presented in Table 2 and Table 3.

### 3.4. Functional and Activity Scores

The Tegner activity scores were similar between the groups preoperatively, with Group A at 7.72 ± 1.65 and Group B at 7.45 ± 2.09 (*p* = 0.076). Postoperatively, Group A had a mean score of 7.28 ± 2.15, whereas Group B had a mean score of 6.79 ± 3.70 (*p* = 0.065). The change in Tegner activity score (Δ) was −0.44 ± 2.76 for Group A and −0.66 ± 4.29 for Group B (*p* = 0.783), with no statistically significant difference between the groups. The mean time to return to sports was significantly shorter in Group A (5.1 ± 1.3 months) than in Group B (7.6 ± 2.1 months) (*p* < 0.01). Overall, 84.61% of the patients returned to their previous activity level. Specifically, 95.2% (20/21) of patients in Group A achieved this outcome, whereas 72.22% (13/18) achieved it in Group B. The preoperative and postoperative Tegner activity scores, as well as Δ values, are shown in Table 2 and Table 3.

### 3.5. Patient Satisfaction

Patient satisfaction was generally high, with 76% (16/21) of patients in Group A reporting being satisfied or very satisfied, compared with 77% (14/18) in Group B. However, three patients in Group B expressed dissatisfaction with the outcome, in contrast to none in Group A.

### 3.6. Complications

No cases of complete patellar dislocation were recorded during the follow-up period in any of our patients. Recurrent patella instability episodes, defined as subluxation of the patella reported by the patient during the follow-up period, were noted in two patients in Group A (9.5%) and three in Group B (16.6%). These patients had mild trochlear dysplasia (Dejour type B). All instability episodes were reported during sporting or cutting activities, without frank dislocation, and the patella was reduced spontaneously. One patient in Group A (4.7%) and two in Group B (11.1%) required knee arthroscopy due to persistent patellofemoral pain. In this patient group, partial cartilage loss of the medial trochlear facet was found, which was treated with debridement, physiotherapy, and kinesiotaping of the patellofemoral joint. There were three cases with a superficial skin infection that were treated conservatively with antibiotics and local wound debridement. There were no patients with leg length discrepancy or knee axis deviation. The complications are summarized in Table 4.

## 4. Discussion

The most important finding of this study is that patients who underwent surgery acutely returned to sports earlier and more often than those with recurrent dislocations. While absolute postoperative PROMs were slightly higher in the acute group (Group A), the magnitude of improvement was greater in the recurrent dislocation group (Group B). This highlights that patients with recurrent dislocation, despite having worse preoperative function, experienced comparable clinical benefits from MPFL reconstruction. Moreover, the anatomical MPFL reconstruction techniques used in the present study were found to be safe and effective for the adolescent athlete population, with a high satisfaction rate and minimal complications.

Although the classic indications for operating on patella instability in children and adolescents are recurrent instability and/or osteochondral fractures, in recent years, there has been a trend in treating acute injuries of the MPFL [11,17,18,19]. In the general population, many systematic reviews have shown that surgical treatment for primary patellar dislocations results in fewer redislocations and better knee function than nonsurgical treatment in the short term [20,21]. In adolescents, comparisons between operative and conservative treatments remain controversial. Two systematic reviews demonstrated the short-term superiority of surgery, whereas one review concluded that both surgical and nonsurgical methods yielded similar outcomes in terms of redislocation and knee function [22,23,24]. Consequently, not all patients respond well to conservative treatment, highlighting the need to establish a risk profile for each individual [25]. In a systematic review by Huntington et al., the risk of recurrent dislocation was found to be greater in younger patients and those with the following factors: open physes, trochlear dysplasia, elevated tibial tuberosity–trochlear groove (TT–TG) distance, and patella alta [4]. The population in our study consisted of highly active athletes or athletic individuals who had at least two of the previously mentioned risk factors. Therefore, our decision to proceed with MPFL reconstruction after the first episode of patellar dislocation can be justified, despite the current trend favoring conservative treatment.

In recent years, interest in MPFL reconstruction has markedly increased. In contrast to adults, surgical treatment in the pediatric population presents significant and technically challenging considerations, particularly in those with an open physis. We used an anatomic MPFL reconstruction technique for femoral fixation, creating a bone tunnel in the distal femur using the Schöttle point as the anatomical landmark or two suture anchors in younger patients. This type of fixation has been shown to be safe, with acceptable complication rates [10]. Although it is well recognized that MPFL reconstruction involving patellar tunnels carries a risk of patellar fracture, no such complication was observed in our series. According to the literature, the incidence of patellar fractures associated with tunnel creation ranges from 0% to 3.6%, with limited distinction among the various surgical techniques. Notably, the risk appears to be higher when blind transverse tunnels are used, as opposed to trans-patellar tunnels [26]. Moreover, given the young age of the study population, we opted for ligament fixation only, avoiding bone realignment procedures. In the literature, isolated MPFL reconstruction is considered a viable first-line surgical option, providing excellent midterm results, regardless of patellar height or trochlear dysplasia [27,28]. In line with this, evidence exists that questions whether all or any of the predisposing factors need to be addressed when performing MPFL reconstruction [29,30].

Regarding the patient-reported outcome measures (PROMs), both groups showed significant improvement. Overall, the results demonstrated that patients who underwent surgery after the first dislocation had significantly better Lysholm, Kujala, Pedi-IKDC, and TAS scores than those in the chronic dislocation group. That being said, while Group A demonstrated higher absolute postoperative PROMs compared to Group B, the magnitude of improvement (Δ) from preoperative to latest follow-up scores appears similar across groups for several outcome measures. The comparable deltas suggest that both groups benefit significantly from surgery, supporting the value of surgical intervention across different clinical presentations. Unfortunately, there are no other studies in the literature comparing MPFL reconstruction between acute and recurrent patellar instability. However, data are available for cases where first-time dislocation is accompanied by a loose body. A recent prospective analysis revealed that conducting concomitant MPFL reconstruction in adolescents with first-time patellar dislocation and an intra-articular loose body results in a fivefold reduction in recurrent instability, reduces the need for additional surgeries, and increases the likelihood of returning to sports compared with repairing or leaving the MPFL untreated [31]. Additionally, our cohort showed that the mean time to return to sports was significantly shorter in Group A than in Group B. Since patellar instability commonly affects young athletes, the primary goal in managing a first patellar dislocation is to achieve functional recovery as quickly as possible, enabling a return to preinjury fitness levels and sporting activity. In this highly demanding subgroup, a delay exceeding two months can result in missed seasons, disrupted team integration, and psychosocial stress associated with postponed participation [32]. Consequently, given that nonsurgical management of first-time patellar dislocation often leads to high recurrence rates and failure to return to sport [33], the results of this study support the benefits of early surgical intervention in appropriately selected patients.

Possible complications after MPFL reconstruction in the pediatric population include redislocation, persistent instability, stiffness, patellofemoral pain, patellofemoral arthritis, and growth disturbance. In our study, although there was no case of recurrent patella dislocation necessitating reoperation, patients from both groups reported patella instability episodes. The overall subluxation rate was 5/39 cases (12.82%)—2/21 cases (9.5%) in Group A and 3/18 cases (16.6%) in Group B. These rates are comparable with those of previous studies, which reported overall recurrence rates ranging from 5% to 13.8% [9,34,35]. However, at this point, it should be noted that among the studies, there is an inconsistency in the definition of recurrence. In our study, instability recurrence was defined as any subluxation episode during the follow-up period. None of the patients in either group experienced complete patellar dislocation after surgery. Moreover, three patients, one from Group A and two from Group B, required reoperation because of persistent patellofemoral pain. Overall, patients in Group A had fewer complications; however, the differences were not statistically significant. Additionally, no cases of growth arrest, limb-length discrepancies, or angular deformities were observed postoperatively throughout the entire follow-up period.

## 5. Limitations

This study has certain limitations. First, the sample size was relatively small, which may have limited the statistical power to detect group differences. Additionally, female patients may have been underrepresented. Anatomical factors such as the tibial tubercle–trochlear groove (TT–TG) distance and patella height (e.g., Caton–Deschamps ratio) were not systematically assessed, which could have influenced the outcomes; however, as discussed, these parameters would not have altered the surgical approach in skeletally immature patients. Another limitation to address is the absence of outcome measures beyond patient-reported outcomes. The inclusion of more objective data, such as strength measurements, would provide a clearer understanding of the functional differences between the two groups. Importantly, the retrospective design introduces a potential for selection and information bias, while the absence of a comparative group treated non-operatively prevents a direct evaluation of surgical versus conservative management. Even if our focus was on operative outcomes, including a non-operative cohort in future prospective studies would provide a more complete picture of the treatment landscape for adolescent patellar instability. Finally, the study sample focused on a highly specific population—adolescent athletes—limiting the generalizability of the conclusions.

## 6. Conclusions

MPFL reconstruction is a safe and effective procedure for both acute and recurrent patellar dislocation in adolescent athletes. While patients who underwent acute reconstruction returned to sport more quickly and showed higher absolute postoperative scores, the greatest overall improvement from preoperative to final follow-up was observed in those treated for recurrent instability. Both surgical approaches demonstrated high satisfaction rates and minimal complications, supporting MPFL reconstruction as a reliable option across varied clinical scenarios.

## Figures and Tables

**Table 1:** Demographic data of the patients included in the study.

		Group A Acute Patella Dislocation	Group B Recurrent Dislocation	*p* Value
**Gender (Male/Female)**		n = 21, 12 males, 9 females	n = 18, 10 males, 8 females	0.72
**Age, years, mean (SD)**		14.7 ± 1.6	14.8 ± 1.3	0.36
**Side (Right/Left)**		13/8	10/8	0.94
**BMI, kg/m^2^, mean (SD)**		23.33 ± 4.12 (21.4–26.4)	24.76 ± 3.08 (20.6–25.9)	0.23
**Follow-up, months, mean (SD)**		39.32 ± 12.1	36.24 ± 16.5	0.51
**Sport of injury, Number**	Football	11	12	
	Basketball	8	5	
	Other	2	1	
**Trochlear Dysplasia, Dejour Grade**	A	16	10	
	B	3	5	
	C	1	3	
	D	1	0	

Abbreviations: SD, standard deviation; BMI, body mass index.

**Table 2:** Preoperative and postoperative Lysholm, Kujala, Pedi-IKDC, Tegner, and VAS scores.

Score	Δ Group A (Acute) Mean ± SD	Δ Group B (Recurrent) Mean ± SD	*p*-Value (ΔA vs ΔB) *
Lysholm	18.39 ± 20.25	28.42 ± 8.62	0.037
Kujala	26.00 ± 22.17	37.44 ± 21.51	0.046
Pedi-IKDC	25.67 ± 13.14	35.46 ± 13.13	0.006
VAS	−2.63 ± 1.66	−3.23 ± 2.49	0.238
Tegner	−0.44 ± 2.76	−0.66 ± 4.29	0.783

* Independent *t*-test, *p* significant at <0.05.

**Table 3:** Change (Δ) in PROMs from preoperative to latest follow-up.

		Group A, Acute Patella Dislocation	Group B, Recurrent Patella Dislocation	
Score				*p* Value
**Lysholm score, mean ± SD**	Preoperatively	74.18 ± 19.23	62.1 ± 11.65	0.01
	Latest follow-up	92.57 ± 6.21	90.53 ± 8.21	0.062
**Kujala score, mean ± SD** **Range**	Preoperatively	68.21 ± 21.01 (48–79)	55.32 ± 17.09 (38–72)	0.01
	Latest follow-up	94.21 ± 9.23 (76–100)	92.76 ± 12.39 (70–100)	0.08
**Pedi-IKDC score, mean ± SD** **Range**	Preoperatively	67.98 ± 12.29 (55–87)	56.2 ± 13.6 (42–74)	0.01
	Latest follow-up	93.65 ± 4.1 (81–98)	91.67 ± 6.21 (75–98)	0.067
**Tegner activity score, mean ± SD** **Range**	Preoperatively	7.72 ± 1.65 (6–9)	7.45 ± 2.09 (6–9)	0.076
	Latest follow-up	7.28 ± 2.15	6.79 ± 3.70	0.065
**VAS score, mean ± SD**	Preoperatively	3.1 ± 1.13	4.21 ± 3.01	0.01
	Latest follow-up	0.47 ± 1.01	0.97 ± 1.32	0.083
**Patient satisfaction**	Satisfied	16	11	
	Very satisfied	4	3	
	Moderate	1	1	
	Not satisfied	0	3	

Abbreviations: Pedi-IKDC, Pediatric International Knee Documentation Committee; SD, standard deviation; VAS, visual analog scale.

**Table 4:** Analysis of complications according to treatment groups A and B.

Complications	Total (n_1+2_/N_1+2_)	Group A (n_1_/N_1_)	Group B (n_2_/N_2_)	*p* Value ^a^
Postoperative subluxation episodes	5/39 (12.82%)	2/21 (9.5%)	3/18 (16.6%)	0.21
Reoperations	3/39 (7.6%)	1/21 (4.7%)	2/18 (11.1%)	0.58

^a^ Chi-square test, *p* significant at <0.05, n_1_= number of cases in Group A, N_1_ = total number of patients in Group A, n_2_ = number of cases in Group B, and N_2_ = total number of patients in Group B.

## Data Availability

The raw data supporting the conclusions of this article will be made. available by the authors upon request.

## Abbreviations

The following abbreviations are used in this manuscript:

MPFL	Medial Patellofemoral Ligament
Pedi-IKDC	Pediatric International Knee Documentation Committee’s scale
VAS	visual analog scale
TAS	Tegner activity scale
TT–TG	Tibial Tubercle–Trochlear Groove
PROMs	Patient-Reported Outcome Measures
